# Breast Cancer Brain Metastases: Clonal Evolution in Clinical Context

**DOI:** 10.3390/ijms18010152

**Published:** 2017-01-13

**Authors:** Jodi M. Saunus, Amy E. McCart Reed, Zhun Leong Lim, Sunil R. Lakhani

**Affiliations:** 1 The University of Queensland (UQ), UQ Centre for Clinical Research, Herston, Queensland 4029, Australia; amy.reed@uq.edu.au (A.E.M.R.); m.lim@uq.edu.au (Z.L.L.); s.lakhani@uq.edu.au (S.R.L.); 2 QIMR Berghofer Medical Research Institute, Herston, Queensland 4006, Australia; 3 Pathology Queensland, Royal Brisbane Women’s Hospital, Herston, Queensland 4029, Australia; 4 UQ School of Medicine, Herston, Queensland 4006, Australia

**Keywords:** breast cancer, brain metastases, clonal evolution

## Abstract

Brain metastases are highly-evolved manifestations of breast cancer arising in a unique microenvironment, giving them exceptional adaptability in the face of new extrinsic pressures. The incidence is rising in line with population ageing, and use of newer therapies that stabilise metastatic disease burden with variable efficacy throughout the body. Historically, there has been a widely-held view that brain metastases do not respond to circulating therapeutics because the blood-brain-barrier (BBB) restricts their uptake. However, emerging data are beginning to paint a more complex picture where the brain acts as a sanctuary for dormant, subclinical proliferations that are initially protected by the BBB, but then exposed to dynamic selection pressures as tumours mature and vascular permeability increases. Here, we review key experimental approaches and landmark studies that have charted the genomic landscape of breast cancer brain metastases. These findings are contextualised with the factors impacting on clonal outgrowth in the brain: intrinsic breast tumour cell capabilities required for brain metastatic fitness, and the neural niche, which is initially hostile to invading cells but then engineered into a tumour-support vehicle by the successful minority. We also discuss how late detection, abnormal vascular perfusion and interstitial fluid dynamics underpin the recalcitrant clinical behaviour of brain metastases, and outline active clinical trials in the context of precision management.

## 1. Clinico-Epidemiologic Profile of Brain Metastatic Breast Cancer

Around 30% of breast cancer (BC) patients develop metastatic (stage IV) disease, and at least 15% will experience symptomatic relapse in the brain [1], a serious complication that causes rapid neurological decline and death in virtually all patients within a few years. Receiving this diagnosis marks a significant downturn for patients, both physiologically and psychologically. In addition to the personal burden, this is also a major socioeconomic problem because it often affects women who are members of the tax-paying workforce, are of childbearing age and/or have dependent children. The direct economic impact is also substantial, with the annual cost of care estimated to be ~$100,000 USD/patient in 2006, more than double stage IV patients without brain metastases (BM) [2]. Young age, high histologic grade, and tumour subtype are major risk factors [3,4]. 

BM are usually confirmed radiologically with magnetic resonance imaging (MRI), with or without gadolinium contrast enhancement, in patients who develop neurological symptoms (e.g., chronic headache, motor, cognitive or speech deficits, and seizures). Symptomatic treatment can include antiepileptic agents (e.g., phenytoin, levetiracetam) and also dexamethasone to control oedema and elevated intracranial pressure. Steroid treatment produces iatrogenic Cushing’s syndrome and contributes to overall morbidity. Depending on the extent of disease, the patient’s age and general health, local control measures can include surgical excision, stereotactic, and/or whole brain radiotherapy (SRS, WBRT). These modalities have been mainstays of treatment since the 1950s, and while their precision and efficacy are improving, ultimately they are not curative. For breast cancer brain metastases (BCBM), a combination of surgery and SRS seemed to provide the most benefit in an historic cohort, increasing median survival to 22 months compared to 5.5 months for WBRT [3]. 

There are currently no chemotherapeutic agents approved by the Federal Drug Administration (FDA) for management of BM. Our understanding of chemotherapy efficacy in different contexts is generally poor (particularly in relation to primary tumour histology and pre-treatment history), due to a lack of prospective clinical trials and drug uptake data. For example, depending on practice standards (which vary internationally and even between centres in the same country), chemotherapy is often given to patients with BM after local measures to control systemic disease, which apparently poses the most imminent health threat—multiple retrospective analyses have shown systemic disease burden is a poor prognostic indicator [5,6,7], and the presence of extracranial mets is a component of the Radiation Therapy Oncology Group’s (RTOG) Graded Prognostic Assessment (GPA) tool [8,9]. In breast cancer, extracranial disease is present in ~80% of patients with BM [10,11], but in contrast to earlier RTOG studies, recent meta-analyses found that extracranial disease is not prognostic after accounting for human epidermal growth factor receptor 2 (HER2) and hormone receptor status [3]. The rationale for chemotherapy treatment is not universal, and prospective data are urgently needed in a more contemporary setting to guide clinical decision-making [12]. 

An American study by Rosner et al. [13] originally demonstrated excellent (by today’s standards) response rates to cytotoxics in 100 consecutive BCBM from a single institution between 1970 and 1985—52%–54% of patients treated with cyclophosphamide, fluorouracil, and prednisone, or methotrexate and vincristine, exhibited partial or complete central nervous system (CNS) responses assessed by computed tomography (CT) imaging. In 1992, Boogerd et al. reported a similarly encouraging response rate of 59% for patients treated with cyclophosphamide, methotrexate and 5-fluorouracil [6]. In contrast to these historic trials, agent selection is now often biased toward those predicted to penetrate the CNS, rather than those predicted to be most efficacious against metastatic breast cancer (expertly reviewed elsewhere [12]). There is evidence that the orally active 5-fluoracil prodrug, capecitabine, is directly effective against BCBM—metabolites can be detected in BM surgical samples from BC patients given a single pre-operative dose [14], objective CNS responses to monotherapy have been observed in a small case series [15] and it enhances the clinical benefit of HER2-targeted therapy [16,17].

## 2. Breast Cancer Cell-Intrinsic Features Can Drive Breast Cancer Metastasis to the Brain

The risk of developing BM is associated with primary tumour phenotype, highest for triple-negative (TN), HER2+, basal-like and claudin-low disease [4,18,19]. While systemic therapy is different between these groups, and obviously impacts BM development, in many cases the primary tumour has already seeded distant organs at the time of initial cancer diagnosis (‘Fisher’s theory’ that cancer is a systemic disease at the time of diagnosis, which originally spurred the move away from radical mastectomy toward breast-conserving surgery with adjuvant treatment in the 1990s [20,21,22]). Micrometastases may also seed other organs (so-called ‘self-seeding’ [23,24]). Therefore, to some degree, brain metastatic fitness is (epi)genetically coded in the primary tumour, influenced by environmental and germline modifiers [25]. Specific examples of this include high expression of HER2, HER3, cyclooxygenase 2 (COX2), heparin-binding EGF-like growth factor (HB-EGF), neuroserpin, neurotrophin-3, and the glycosyltransferase ST6GALNAC5; and suppression of *PTEN* [26,27,28,29,30,31].

The requirements for initially colonising the brain are fundamentally different to those needed for sustained outgrowth, however some changes associated with risk of brain relapse have also been identified in BM surgical samples, implying a continuing requirement for outgrowth in the brain. For example, there are now robust data from independent studies showing that *ERBB2* over-expression and *PTEN* suppression in BM are underpinned by positive selection of hard-wired DNA copy-number alterations (CNAs) [29,30]. Other genes harbouring CNAs with corresponding changes in expression have recently been identified, but their significance remains to be elucidated. For example, the mitochondrial protein *TMEM65* is amplified and overexpressed in ~50% of BM from breast and lung cancers, *SOX11* is amplified and overexpressed in ~30% of breast and ~80% of lung cancer-BM, and *NRG1* is lost and suppressed in ~60% of BCBM [29,32]. Large BC genome sequencing projects have not yet illuminated particular mutant alleles that bestow brain metastatic fitness, but hopefully these analyses will be forthcoming with the assembly of additional clinical data that includes site-specific relapse information.

Pre-programming of metastatic behaviour is also mediated systemically by tumour-derived microvesicles that circulate miRNA, mRNA and protein cargoes throughout the body. Exosome cargoes can prime pre-metastatic niches to receive circulating tumour cells (CTCs) and create a favourable niche for their outgrowth. Mechanistically this can involve vascular permeabilisation, angiogenesis, and extracellular matrix remodelling [33,34,35]. Molecular profiling of exosomes from brain-seeking cell lines have implicated particular miRNA and protein cargoes, but the clinical significance of these candidates is yet to be determined [36]. With the exception of elegant experiments showing that exosomes carrying integrin-β3 can mediate brain-tropic behaviour [37], little is known about microvesicle specification of brain relapse. In particular, the field eagerly awaits technological developments that will facilitate microvesicle profiling from prospectively-collected patient blood samples.

BM are usually associated with extracranial metastases, particularly the liver, as the two are likely trapping sites for circulating tumour cells (CTCs), but 10%–17% of BM are not associated with extracranial disease [10,11,38] (significantly less frequent in African-American patients [39]). The relationship with clinical outcome is uncertain—brain-only-metastasis (BoM) has been associated with both poor and favourable survival relative to other patterns of disease spread [11,38]. Interestingly, BoM is not linked to HER2 or hormone receptor status [10], but is associated with expression of HER3 [27], which is the preferred oncogenic dimerization partner of HER2, and has ample access to neuregulin ligands in the brain [40,41,42]. Molecular profiling of BCs that exhibit BoM may identify new mechanisms and clinically informative biomarkers. Overall the frequency of BCs exhibiting BoM is only 14–25 for every 1000 cases, and so assembling a sample cohort to look for (epi)genetic features associated with this behaviour could be logistically challenging, however the clinical homogeneity in this group may enable molecular discoveries using a relatively small cohort.

## 3. Extrinsic Factors That Drive Clonal Evolution in Brain Metastases

### 3.1. Microenvironment-Driven Selection Pressure

**Figure 1:**
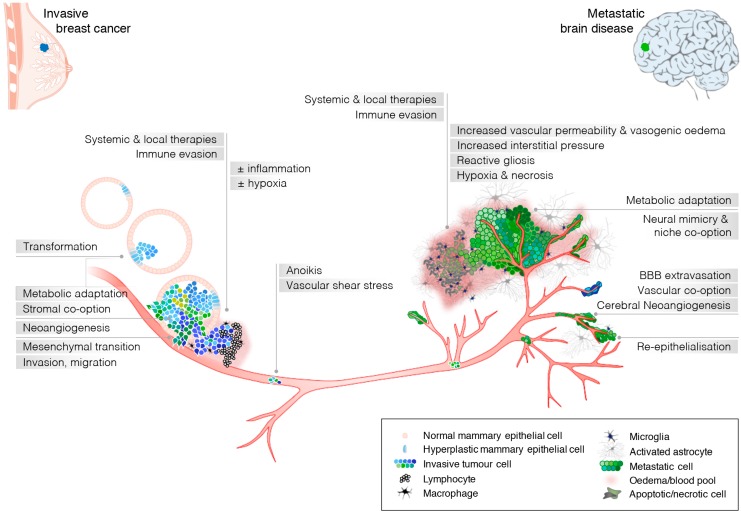
Schematic showing the breast cancer brain metastatic cascade; Requisite capabilities for metastatic fitness and extrinsic pressures driving clonal evolution are indicated in horizontal and vertical tracks, respectively.

The development of BM from CTCs is an incredibly complex process featuring continuous extrinsic selection pressure (depicted in Figure 1). This is initially driven by a requirement for CTCs to circumvent anoikis, tolerate shear stress in the circulation, then extravasate at distant sites, which ultimately favours cells most capable of co-opting the distant niche. Detailed analysis of BC patient CTC-derived cell lines implicated HER2, epidermal growth factor receptor (EGFR), heparanase (HPSE) and Notch1 in brain metastatic fitness [43]. Progression depends on actively crossing the blood-brain-barrier (BBB), the specialised vascular lining separating neural and vascular compartments of the central nervous system. The BBB features continuous tight junctions that oppose paracellular diffusion, diverting the passage of circulating metabolic substrates, xenobiotics, and other solutes to selective transcellular transport systems. Its function depends on contact between endothelia, the vascular-astroglial basement membrane, pericytes and the glia limitans, an interconnected layer of astrocyte foot processes that is key to neurovascular regulation [44]. Ultrastructural and immunofluorescent imaging data show that a fraction of CTCs arrest in capillaries or venules [45], and occasionally extend filopodia that mechanically push through the endothelium and adhere to the basement membrane [46,47,48]. Filopodia then dynamically extends and retracts into the parenchyma as cells migrate to a perivascular position [46,48]. Mediators of this process include specific adhesion molecules and proteases (e.g., integrins α5, β1, β3, cathepsin-S, matrix metalloproteinases (MMPs), and E-selectin [47,49,50,51,52,53]). 

The perivascular niche (PVN) is another critical point of tumour cell attrition—most are unable to tolerate the neuroinflammatory reaction that is rapidly instigated by microglia and astrocytes [28,45,48,54,55,56,57,58,59,60,61]. Cross-talk with resident pericytes and endothelia in the PVN seems to be key for tumour cell survival, quiescence, and therapeutic resistance—capabilities that underpin dormancy [62]. Further progression can take months or years depending on the evolutionary distance between BM-competent cells and their ancestors, and also their inherent adaptability. For example, aberrant DNA repair is common feature of BM, likely an adaptation to oxidative stress [63]. DNA repair is also defective in triple-negative breast cancer (TNBC), which relapses in the brain earlier than other subtypes [64], suggesting that pre-existing DNA repair defects could underlie rapid clinical progression. In terms of ‘late metastasising’ BCs, the anti-angiogenic glycoprotein thrombospondin-1, secreted by endothelia lining stable microvessels, has been linked to dormancy [62]. Inducing neo-angiogenesis could be a key milestone for tumours escaping dormancy, as endothelial tip cells are a source of tumour-promoting transforming growth factor β1 (TGF-β1) and periostin that seem to overcome the effects of thrombospondin. 

In order for micrometastases to progress, the initially hostile neural niche must be transformed into one that promotes colonisation, which essentially involves transforming the glial compartment into a tumour support engine [52,54,65,66,67]. Cells succeeding to this stage seem to migrate along paths of least resistance, dividing as they go. Some proliferate in the perivascular pathway, forming sheaths as they co-opt the vasculature; others prefer interstitial tracks and form well-demarcated parenchymal lesions [28,68]. These patterns co-exist in any given tumour at different proportions [69], but whether achieved by co-option or angiogenesis, vascular proximity is critical for outgrowth [48,70]. 

Tumour cells use remarkable mechanisms to co-opt the neural niche. They cope with oxidative stress, repurpose neurotransmitters as metabolic substrates, recruit and promote the differentiation of neural progenitors into astrocyte support cells, mimic neural traits and effectively ‘plug-and-play’ with the new niche by inducing growth factor receptors (e.g., HER3, HER4, NTR3), particularly those that converge on the akt/PI3K/mTOR, mitogen-activated protein kinases (MAPK) and NF-κB [27,29,54,63,65,71,72,73]. Loss of the phosphoinositide 3-kinase (PI3K) inhibitor *PTEN* is one of the only recurrent genomic features identified to date [29,30,72], but a recent pioneering study by Zhang et al. [67] demonstrated that *PTEN* suppression can also occur as a reversible adaptation. They found that astrocyte-derived exosomes with cargoes from the miR-17/92 locus were able to silence *PTEN* in micrometastatic cells, which increased their secretion of microglia-activating C-C motif chemokine ligand 2 (CCL2) and promoted outgrowth [67]. 

### 3.2. Therapy and Clonal Selection

Historically, there has been a widely-held view that uptake of circulating drugs is severely limited by the BBB, since BM arise on a background of pre-treatment, and chemotherapeutics accumulate to sub-efficacious concentrations in experimental BM. For example, using fluorescence and phosphorescence brain imaging in intravenously xenografted mice and 4T1 syngeneic BM, Lockman et al. [74] found that vascular integrity was compromised in 89% of BM, but uptake of paclitaxel and doxorubicin was heterogeneous and up to 30-fold lower than extracranial tumours, reaching cytotoxic levels in just 10% of the most permeable BM [74]. Analysis of lapatinib produced similar results, with variable and low uptake, and no evidence of intrinsic resistance in ex vivo BM cell cultures from treated mice [75]. However, the idea that insufficient uptake across the BBB is a universally limiting factor in human patients is at odds with several key lines of evidence. Firstly, detection of BM with contrast-enhanced MRI works on the basis that contrast agents move passively from the vascular space to the interstitium through vessel fenestrations, which occurs in BM larger than ~5 mm—so contrasting lesions are inherently ‘leaky’. Secondly, CNS response rates of >50% were observed in early chemotherapy trials [6,13], despite the use of hydrophilic agents that do not readily transverse intact vasculature, suggesting mouse models may not adequately recapitulate human BM tissue architecture or pharmacokinetic-pharmacodynamic determinants of drug efficacy. Finally, PET imaging studies have shown that large monoclonal antibodies (mAbs) accumulate in human BM to levels associated with efficacy at other sites [76,77,78,79,80], indicating that the treatment-refractory behaviour of BM is likely due to more than permeability alone [77]. Importantly, the distribution and cellular uptake of non-targeted therapies are passive and nonspecific, ultimately determined by each agent’s molecular structure, fluid gradients, and rates of metabolism and clearance. Vascular perfusion, intracranial pressure and interstitial fluid dynamics are abnormal in BM, and collectively oppose passive drug diffusion (Section 5.2), whereas tumour-specific mAbs generally have longer half-lives and accumulate in tumour deposits. Studies correlating vascular permeability, fluid flow dynamics, tumour interstitial pressure and drug uptake in human BM would be very informative in terms of considering the impact of BM-specific physiologic factors on drug dosing and efficacy.

We tend to relate clonal progression to clinical progression directly, on a patient or whole-tumour scale, but cycles of seeding, dormancy and regression are likely occurring simultaneously throughout the cerebrum on staggered timelines. BM arise from convergence of perivascular and interstitial proliferations, including index/parent lesions as well as the progeny of tumour self-seeding, all encased in reactive brain parenchyma [55,81]. As small lesions that were once fully protected behind an intact BBB become increasingly permeable, for a time they may be exposed to sub-efficacious drug concentrations, providing an ideal milieu for selection and outgrowth of resistant clones. In this context, another important consideration is that micrometastases may be effectively naïve to prior systemic therapy if protected behind an intact BBB segment at the time of initial treatment, and may respond to treatment once vascular permeability increases. Consistent with this idea, Boogerd et al. [6] reported clinical and objective CNS responses of 88% and 62% in a small cohort of BCBM patients treated with cytotoxic therapy in the late 1980s, including 100% (7/7) and 70% (5/7) who had previously been treated with the same regimens in the adjuvant setting or for progressive systemic disease [6].

Identifying the capabilities that are clonally selected in BM arising on a background of pre-treatment could inform the development of more holistic therapeutic strategies. For example, a landmark preclinical study from the Massagué team showed that protocadherin 7-expressing tumour cells form gap junctions with astrocytes, creating a feedback situation where cGAMP passed to astrocytes induce secretion of interferon α (IFNα) and tumour necrosis factor (TNF). In turn, the cytokines act as paracrine factors that promote tumour growth and chemoresistance through signal transducer and activator of transcription 1 (STAT1) and NF-κB [61]. These findings prompted a clinical trial exploring the feasibility of using the gap junction modulator, meclofenamate, for BM treatment (Table 1). Another study assessing the efficacy of PI3K/mTOR therapy in HER2+ BM patient-derived xenografts (PdX) found that non-responders exhibited higher genomic instability and defective DNA repair compared to responders [72], consistent with a separate report on mTOR treatment resistance [82]. These findings raise the possibility of using poly (ADP-ribose) polymerase (PARP) inhibitors to chemosensitise HER2+ BM with hypermutator phenotypes. There is also circumstantial evidence implicating HER3 in drug resistance—it is induced and activated in BM from breast and lung cancers, and has been separately implicated in resistance to anti-oestrogen, -HER2 and cytotoxic therapies [83,84,85,86,87]. Antibodies targeting HER3 (e.g., patritumab, AV-203, MM-121, AMG888, and HER2/3 bi-specifics) are currently being assessed for treatment of various solid cancers, though so far, not specifically BM. Molecular profiling of experimental or clinical samples representing pre- and post-therapy clonality and transcriptome profiles is urgently needed to identify additional candidates.

## 4. The Molecular Portrait of Breast Cancer Brain Metastases

An increasing number of studies are reporting high-resolution analysis of clinical and experimental samples to address biological questions and clinical challenges associated with BCBM. Key approaches, their strengths, limitations, and landmark findings are discussed below (Table 2).

### 4.1. Analysis of ‘Brain-Seeking’ Clonal Cell Line Derivatives

To enrich for brain-tropic (epi)genomic traits, BC cell lines can be intravenously injected into experimental mice, developing BMs isolated, expanded in vitro, and subjected to successive in vivo passage cycles. Brain-tropic cell line derivatives have been applied in various ways, including the comparison to more heterogeneous parental cultures to identify genomic/transcriptomic traits associated with BM. Most commonly used are: the TNBC line, MDA-MB-231 and its brain-tropic derivative MDA-MB-231_Br; the syngeneic mouse 4T1.2 pair, also triple-negative; the BT-474 pair (HER2+) and HER2-overexpressing MDA-MB-231_Br cells, which produce BM of approximately three-fold greater size than the parental line [63,74,89,97,98,99]. While these lines don’t fully recapitulate the phenotypic diversity of brain-tropic BC, they do provide functional experiments with greater relevance than lines derived from primary breast tumours or pleural effusions, and also guarantee higher take rates when generating experimental cohorts. Brain-seeking derivatives of five BC cell lines and the parental lines have been exome profiled [100], with little evidence found for a definitive genetic driver. Indeed, Jacob et al. [100] report that metastatic fitness can arise without de novo mutation, and can simply occur from further enrichment of particular mutant alleles.

The pioneering investigation of gene expression profiles in brain-seeking derivatives of two lines originally derived from TNBC pleural effusions (MDA-MB-231 and CN34) identified that *COX2*, *HBEGF*, and *ST6GALNAC5* are enhancers of BBB extravasation [89]; COX2 and HBEGF, but not ST6GALNAC5, also likely function in extravasation in the lung. Interestingly, these genes were not differentially expressed in an independent cohort [101], nor could the role of ST6GALNAC5 in BBB extravasation be verified in a separate study [102]. There could be a technical or sampling-related basis for these discrepancies; for example, the independent studies exclusively analysed TNBCs while the original used unselected cases, and the BBB model was inherently different, comprising haematopoietic stem cell-derived endothelia and pericytes rather than umbilical vein endothelial cells and astrocytes. 

These inconsistencies do, however, highlight some important limitations of model systems (extensively reviewed elsewhere [103]). As with any xenograft, a major limitation is that the brain-seeking system involves a mouse host supporting human cells in the absence of a full immune complement, a crucial impediment to the usual spread of cancer. On the other hand, syngeneic systems (e.g., 4T1.2) that model innate anti-tumour responses are also flawed because multiple aspects may not be representative of human biology/physiology. The in vitro expansion steps in between successive in vivo passages also impart a selection pressure not encountered in vivo, and intra-tumoural heterogeneity is reduced in these models. Nonetheless, brain-tropic cell lines are reproducible models amenable to controlled hypothesis testing, and are very important experimental tools for BM research. 

### 4.2. Analysis of Human Clinical Samples

High-resolution molecular profiling of human tumours is essential to ensure relevance and make discoveries that lead to clinically translatable outcomes. Technological improvements have reduced the cost and vastly increased the volume and scale of cancer (epi)genome and transcriptome analysis. While The Cancer Genome Atlas (TCGA) and International Cancer Genome Consortium (ICGC) have made huge advances cataloguing BC genomes [104,105,106], currently they are not focussing on metastatic deposits. BM resection is not routinely performed and, thus, fresh frozen tissue samples are rare, however, prospectively collected cohorts are beginning to be sequenced by independent groups, and methodological advances are now allowing analysis of the lower quality, fragmented DNA and RNA from more accessible formalin-fixed, paraffin-embedded (FFPE) samples. Considering the vast cohort sizes and volumes of genomic data generated for primary BC [104,105,106], much of which is on the precipice of clinical translation, there is significant scope for extending the depth and breadth of (epi)genomic and transcriptomic analysis of BM.

A plethora of ‘omics studies with small BM cohorts and/or targeted gene panels have emerged in the last few years (Table 1 [30,32,91,92,94,95,96]). Unsurprisingly, genes frequently altered in BC were also commonly detectable in BM, though mutant allele frequencies (MAF) for some were enriched in BM (e.g., *TP53*, *PTEN*, *ATM*) [30,32,94]. The high frequency of potentially actionable mutations identified supports implementation of BM diagnostic profiling to inform targeted therapy. Interestingly, a ‘BRCA1-deficient-like’ signature was identified in HER2+ BM with wild-type *BRCA1*, raising the possibility of using PARP inhibitors [91]. Indeed, inipirib and veliparib are being assessed clinically (Table 1). These trials are focusing on TNBC or unselected BC, but prospective assignment of HER2+ cases to PARPi therapy based on molecular profiling of any previously resected BM would be a rational next step to see whether the signature has predictive power in this group.

In terms of illuminating aspects of the biology, novel candidates have been identified in clinical sample discovery cohorts and validated, or are awaiting functional investigation in the field (Table 2). These include altered genes with corresponding changes in expression, functional gene networks with members collectively mutated more frequently than expected by chance, or that are differentially expressed between BM and matching primary tumours (Section 4.3). A unifying observation from the sequencing of primary tumour genomes is that cancer is underpinned by vast heterogeneity, with a seemingly infinite number of roads leading to Rome. However, in metastatic disease the distant ‘host organ’ is a common denominator, and so based on the idea that the neural niche may surpass intrinsic alterations in BM with different histologies and treatment histories, our group profiled the genomic and transcriptomic landscapes of BM from melanoma, lung, and breast cancers [29]. We integrated the data to identify recurrently altered, functionally-interconnected, gene networks (DNA repair, axon guidance, HER/*ERBB* and protein kinase-A signalling), and individual genes harbouring an unusually high number of expressed mutations that were predicted to be deleterious (e.g., *DSC2*, *ST7*, *PIK3R1*, *SMC5*). 

The main limitations of using clinical samples in this way are, firstly, a lack of full control over other variables like patient age, germline modifiers, and treatment history. Secondly, sampling bias—profiling is usually performed on small pieces of larger tumours, which may not be representative of the whole tumour. Using larger amounts of tissue to circumvent sampling bias necessitates sequencing with more depth (thus more cost) in order detect subclonal alterations. Finally, clonal diversity reflects the growth requirements at the time of surgical excision, and so alterations that facilitate earlier stages of metastatic progression may be heavily diluted once selection pressure wanes—profiling human BM is essentially an endpoint analysis biased toward the latest stages of the metastatic cascade.

### 4.3. Subtractive Analysis of Breast Cancer-Brain Met Pairs

Comparative analysis of patient-matched pairs of primary tumour and normal tissue is essential to determine whether genetic variations are somatically acquired or inherited, and a ‘trio’ that also includes matching metastatic deposits is often considered the pinnacle for identifying metastasis-associated changes. In 2010, Ding et al. profiled the exomes and CNAs of a BM, primary tumour and a PdX model, and showed that BM are seeded from a minority of primary tumour cells [90]. The tumours were closely related, with 48/50 variants present in all three lesions. There was extensive heterogeneity in the primary, but a reduced MAF range in the BM and PdX, reflecting the selection processes involved in metastatic progression. Brastianos et al. recently sequenced the exomes of 86 trios, achieving impressive statistical power for deep analysis of clonal selection [93]. The majority of cases exhibited branching evolution, with a ‘trunk’ of shared changes and a series of ‘private’ mutations reflecting continual independent evolution. Alterations predicting sensitivity to PI3K/AKT/mTOR, CDK and HER2/EGFR inhibitors were identified and, importantly, in 53% of patients, these clinically informative or targetable mutations were not detected in the primary tumours. While extracranial disease deposits (e.g., lymph node mets, pleural effusions) may be more accessible for biopsy than BM, these were also highly divergent. These deep genomic data extend other findings from matched metastatic deposits (Table 2), which collectively indicate that management decisions should be based on BM diagnostic profiling wherever possible, rather than primary tumour or extracranial disease biomarkers. 

Matched clinical sample pairs are often only available as archival specimens, with severely fragmented RNA. However, specialised technologies have been successfully applied to identify candidate mediators of BM development. cDNA-mediated annealing, selection, extension and ligation (DASL) expression array profiling on 39 matched pairs revealed *ERBB3* (HER3) and its adaptor protein *GRB7* amongst genes most significantly induced in BM [73]. HER3 induction and activation have since been confirmed in independent, matched BM cohorts from breast and lung cancers, and unmatched BM from a wide range of primary cancer types, suggesting this is an adaptation of carcinoma cells to the neuregulin-rich microenvironment [29,73,107,108]. Others found that double-strand DNA damage repair genes (including *BARD1* and *RAD51*) were over-represented in the BM compared to matching BCs [63], and in a third example, gene expression (GEX) profiling of eight BM compared to unpaired but clinically matched BC found that overexpression of hexokinase 2 (*HK2*) was associated with poor survival [109]. 

Anecdotally, there is often an expectation that identifying private alterations and differentially expressed genes will reveal the (epi)genetic history of metastatic disease and illuminate the biology underpinning progression, because the first studies of this kind were revolutionary (e.g., [73,89,90]), and according to classical scientific method, the most robust hypothesis testing involves test and control. However, even the ostensibly ‘controlled’ design of matched pair/trio studies is limited by unavoidable issues that confound interpretation of any tissue profiling experiment—sampling bias, and the ‘endpoint’ nature of the analysis. Notwithstanding the insights that subtractive approaches can provide into the biology involved, they discount candidates that may have important roles at both primary and metastatic sites, and the candidates identified are not necessarily clinically relevant, as primary BCs are usually successfully treated months or years before clinical presentation of brain disease.

## 5. Factors Underlying the Recalcitrant Behaviour of Brain Metastases

### 5.1. Late Detection

Currently, surgical stump recurrence is common because BM are poorly demarcated, excising a margin of normal brain tissue is not appropriate, and the residual cells that persist in the post-irradiation tissue environment are very pervasive [110]. There is an urgent need for effective molecular-targeted therapies to augment local control measures [77,111], but prospective clinical data for established BM are lacking. This is largely because historically, a heavy co-morbidity burden and poor prognosis restricted participation of BM patients in clinical trials. Where they were included with no/minimal impact on intracranial disease progression, this was often interpreted as an overall lack of efficacy, yet we know that dosing is critical for achieving optimal delivery [112], and that the brain microenvironment impacts substantially on uptake and efficacy [12,75,77,111]. Some trials have assessed brain relapse as a secondary endpoint (e.g., [113]), though were essentially assessing prevention, or efficacy against small, asymptomatic BM with intact microvasculature. With time, modification to traditional clinical trial design [114,115], and recognition that BM are not completely impenetrable to circulating agents, this trend is beginning to change (Table 1).

Recurrence also occurs at new sites in the brain. Deep exome sequencing has shown that, in any given patient, there is far less divergence amongst consecutive BM than between BM and matching primary or extracranial tumours [93], consistent with new lesions arising from stochastic awakening of dormant micrometastases, and/or self-seeding. Even synchronous BM that presented months apart were found to be close relatives despite treatment in the interim. This indicates that the next level of resistance was attained with minimal additional genomic change (through epigenomic or non-coding alterations not captured by exome sequencing); and perhaps also reflects that the requisite changes can be dynamic, reversible adaptations mediated via microenvironmental cross-talk [67,116]. It is becoming clear that the role of the brain microenvironment in driving therapeutic resistance has been severely underestimated [77,117]. For example, while neither trastuzumab ± pertuzumab therapy reduces the incidence of BM, these treatments delay BM development, reflecting a window of time in which resistant clones grow out after an initial debulking effect [113,118].

In any case, by the time they are symptomatic and detected clinically, BM are essentially highly evolved manifestations of BC that have already developed resistance to multiple lines of therapy, and can efficiently adapt to new extrinsic pressures. So long as they are identified at such a late stage, any treatment benefits will continue to be incremental [119] and yet, currently, diagnostic imaging is routinely performed on symptomatic patients and not for screening purposes. The risk of BM is increasing with improving systemic disease control [2,113,118,120,121], indicating that micrometastases can develop into clinically significant BM if given time and opportunity. Critically, while 14%–15% of breast cancer patients develop symptomatic BM, evidence suggests they are actually far more prevalent—in a meta-analysis of CT/MRI data acquired to satisfy eligibility criteria for anti-angiogenic therapy trials in the late 1990s, Miller et al. found a roughly equal proportion of BC patients (~15%) had clinically occult BM [122]. Systematic histopathologic analyses of tissues taken at autopsy suggest the true incidence could be as high as 30%–40% [123,124,125]. Additionally, longitudinal imaging in mice has shown that micrometastases are substantially more prevalent than overt BM, while elegantly highlighting how mouse models can be applied to study metastatic dormancy (expertly reviewed elsewhere [126]). If metastatic screening is to be considered in the future to see if we can treat small and/or dormant lesions with curative intent, we need to couple the transition to precision cancer care with a search for reliable diagnostic imaging targets [127], develop clever conjugates that can cross the BBB to access small, dormant deposits with intact microvasculature, and develop prognostic markers to identify high-risk patients for whom metastatic screening is warranted.

### 5.2. Abnormal Vascular Perfusion and Hypoxia Leads to Inadequate Drug Uptake and Therapeutic Resistance

In the absence of adequate drug uptake data from human BM [14,128], animal modelling has led the field to coalesce on a view that while the BBB is leaky, it is not leaky enough to permit the uptake of systemic therapy to efficacious concentrations. While heterogeneous BBB permeability is most certainly one component of the clinical challenge, the situation is likely far more complex, with vasogenic oedema and elevated intracranial pressure also acting as critical determinants of drug bioavailability. Ongoing proliferation in solid tumours can fuel a perpetual cycle of hypoxia and unchecked neo-angiogenesis, creating chaotic, dysfunctional microvascular networks [129]. The constant vascular remodelling results in dyscoordinated vasoregulation, abnormal hydrostatic pressure gradients, and blood flow patterns, which, paradoxically, creates areas of sluggish blood flow and poor drug penetration in an otherwise hypervascularised environment [45,129,130]. These factors collectively underpin drug delivery challenges in BM, some of which are common to solid tumours, while others may be unique to the brain microenvironment. 

Abnormal fluid dynamics also reduces drug efficacy because it leads to patchy hypoxia. Radiotherapy and certain cytotoxics act by generating reactive oxygen species that damage DNA, but strand breaks are more readily repairable in the absence of oxygen, allowing cells to escape fatal chromosome aberrations and, instead, erroneously repair DNA to increase genetic diversity. Hence, there are strong links between tumour hypoxia, cancer stem cell activity and chemo-/radio-resistance [131]. Indeed, BM are characterised by abnormal DNA repair, and hypermutator phenotypes have been associated with drug resistance in PdX of BM [63,72,91]. 

## 6. Future Directions and Final Comments

Given that inadequate and uneven delivery are critical factors limiting drug penetration, the field is beginning to explore alternative modes of delivery other than classic intravenous supply of naked compounds [132]. For example, several clinical trials are attempting to chemo/radiosensitise BM by targeting neoangiogenesis with VEGF signalling inhibitors (e.g., bevacizumab, sorafenib; Table 1). The intended effect of anti-angiogenic drugs was originally to starve tumours of oxygen but, in the context of combination regimens, blocking development of immature vessels may be beneficial because it reduces vascular tortuosity, normalises perfusion dynamics and increases oxygenation, thus enhancing drug delivery and the efficacy of DNA-damaging agents—the so-called ‘vascular normalisation’ effect [129,133]. 

Other innovative approaches being investigated include carrier- or receptor-mediated drug transport across the BBB [132], and injected microbubbles that oscillate and permeabilise the BBB when energised using anatomically focused, MRI-guided ultrasound [134,135], which could improve drug bioavailability and improve immune recognition. Others are focusing on the development of multifunctional nanoconjugates designed to overcome multiple biological and physiological barriers before releasing their therapeutic payloads [136,137,138]. This is a very attractive concept for cancer therapy generally, but particularly for brain tumours where the BBB represents a unique challenge. Moreover, when it is not feasible to obtain a tissue biopsy to inform management decisions (e.g., high comorbidity burden, oligometastatic disease, or inoperable anatomical location), nanoconjugates could provide unprecedented theranostic capabilities [139].

**Figure 2:**
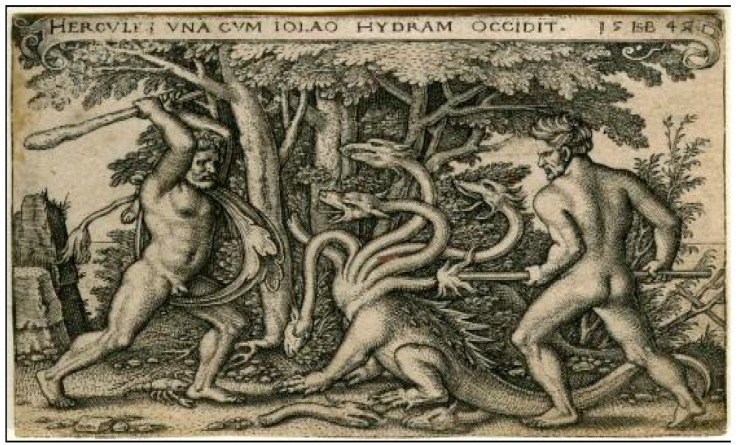
Depiction of the ancient Greek/Roman divine hero, Heracles, and his nephew (Iolaus) fighting the Hydra of Lerna, a serpentine water monster with heads that regenerated stronger and more numerous if severed [142].

In ancient Roman and Greek mythology, the Lernean Hydra was a serpentine water monster, raised by the goddess Hera to kill her illegitimate stepson and son of Zeus, Heracles. Hydra had toxic breath, multiple heads with venomous fangs, and grew new heads for every one severed by its opponents (Figure 2). In a way, this fable is a good analogy for metastatic brain disease, symbolising the perceived hopelessness of challenging an apparently immortal beast that becomes more empowered with every attempt to decapitate it. In the end, Hydra was defeated by Heracles, who used the serpent’s own poisonous blood to burn each severed head so it could not regrow. Numerous strategies have been proposed to improve the clinical management of BC patients with metastatic brain disease, including prevention; better risk prediction and diagnosis of small, more manageable BM using molecular imaging; targeted drug delivery vectors, like nanoparticles; application of targeted agents to the neurosurgical cavity and externally activating sites of bioavailability to targeted drug conjugates (e.g., ultrasound BBB permeabilisation). Taking the Hydra analogy further, one idea gaining more support in the biomedical community is simultaneous targeting of tumour cell abnormalities and features of the neural niche on which growth and drug resistance depend [77,140,141]. Multiple studies have now verified the role of the metastatic brain tumour microenvironment in driving adaptation, outgrowth and drug resistance [29,54,57,65,66,67,116]. A noteworthy example of how these findings are being clinically translated is an ongoing meclofenamate feasibility study (a nonsteroidal anti-inflammatory drug (NSAID) traditionally used for treatment of pain, but which also inhibits gap junction gating). The community eagerly awaits similar examples in the future. 

## Figures and Tables

**Table 1:** Summary of current clinical trials of molecular-targeted agents for breast cancer patients with established brain metastases [88].

NCT-ID	Subtype	Phase	Experimental Arm(s)	Comparator Arm	Approach	Primary Endpoints
02429570	All	0	Meclofenamate	NA	GAP junction modulator	ORR, PFS, safety
01621906	All	0	WBRT + Sorafenib + [18F]FLT PET at baseline	WBRT + [18F]FLT PET at baseline	XRT + VEGFR	RR (radiographic)
01386580	All	1/2	Glutathione pegylated liposomal doxorubicin	Glutathione-pegylated liposomal dox + Trastuz	Carrier (CTx + HER2)	MTD, safety
01132664	HER2+	1/2	Buparlisib + Trastuz	Buparlisib + Trastuz + Capecitabine	VEGFR + HER2 + CTx	MTD, RR, PFS, safety
02154529	HER2+	1/2	Tesevatinib + Trastuz	Tesevatinib dose escalation + Trastuz	Broad-spec RTKi	MTD, PFS, RR, safety
01921335	HER2+	1	ARRY-380 + Trastuz	ARRY-380 dose escalation + Trastuz	HER2	MTD, RR and PFS
01332929	All	1	Bevacizumab + WRBT	Bevacizumab dose escalation + WRBT	XRT + VEGFR	MTD, RR, PFS
02598427	HER2+	1	Intrathecal Pertuzumab + Trastuz	Pertuzumab dose escalation + Trastuz	HER2 (CSF delivery)	MTD, safety
02650752	HER2+	1	Lapatinib + Capecitabine	Lapatinib dose escalation + Capecitabine	CTx + HER2	MTD, RR, PFS
01276210	All	1	Sorafenib tosylate + SRS	Sorafenib tosylate dose escalation + SRS	VEGFR + Raf kinase	MTD, RR, PFS
00981890	All	1	Sunitinib + SRS	NA	XRT + VEGFR	Safety, MTD
00649207	All	1	Veliparib + WBRT	Veliparib dose escalation + WBRT	PARPi	MTD, safety
01724606	All	1	Sorafenib + WBRT	Sorafenib dose escalation + WBRT	XRT + VEGFR	MTD, safety
02308020	All	2	Abemaciclib	NA	CDK4/6i	RR, PFS, safety
02768337	All	2	Afatinib + 4 Gy XRT	Afatinib	XRT + HER2	Drug uptake
01441596	HER2+	2	Afatinib + vinorelbine	Afatinib	CTx + HER2	PFS
02048059	All	2	ANG1005 (formerly GRN1005)	NA	Carrier (CTx)	RR, PFS, OS
01898130	All	2	Bevacizumab	NA	VEGFR + HER2	RR, PFS, safety
02000882	All	2	Buparlisib + Capecitabine (+Trastuz if HER2+)	NA	CTx + panPI3Ki	RR
01934894	HER2+	2	Cabazitaxel + Lapatinib	Cabazitaxel + Lapatinib (different doses)	CTx + HER2	RR, MTD, safety
02260531	All	2	Cabozantinib + Trastuz	Cabozantinib	c-met + VEGFR	RR, PFS, safety
02669914	All	2	Durvalumab (MEDI4736)	NA	PDL1i	RR, PFS, safety
01305941	HER2+	2	Everolimus + Vinorelbine + Trastuz	NA	CTx + HER2	RR, PFS, safety
01480583	HER2+	2	GRN1005 + Trastuz	GRN1005 alone	Carrier (CTx + HER2)	RR, PFS, safety
01494662	HER2+	2	Neratinib (HKI-272)	Neratinib (HKI-272) + Capecitabine	CTx + HER2	RR, PFS, safety
01173497	TNBC	2	Iniparib + Irinotecan	NA	CTx + PARPi	Efficacy, RR
01783756	HER2+	2	Lapatinib + Everolimus + Capecitabine	NA	CTx + HER2 + mTORi	RR, PFS, safety
01622868	HER2+	2	Lapatinib + WBRT or SRS	WBRT or SRS	XRT + HER2	RR, PFS, safety
01218529	All	2	Lapatinib + WRBT	NA	XRT + HER2	RR
02614794	HER2+	2	ONT-380 + Capecitabine + Trastuz	Placebo + Capecitabine + Trastuz	CTx + HER2	PFS, RR, safety
02774681	All	2	Palbociclib (+Trastuz if HER2+)	NA	CDK4/6i	RR (radiographic), PFS, safety
02312622	All	2	Pegylated irinotecan (NKTR 102)	NA	Carrier (CTx)	Disease control rate, PFS
02536339	HER2+	2	Pertuzumab + Trastuz	NA	HER2	RR, PFS, OS, safety
01924351	HER2+	2	SRS + HER-2 directed therapy	NA	XRT + HER2	Relapse rate
02571530	HER2+	2	Intra-arterial cerebral infusion of Trastuz	May consider dose escalation	HER2	MTD, OS, PFS
00303992	HER2+	2	Trastuz + Irinotecan	NA	CTx + HER2	RR, disease progression
02185352	All	2	WBRT + Bevacizumab, Etoposide, Cisplatin	WBRT alone	XRT + VEGFR	RR, PFS
00820222	HER2+	3	Lapatinib + Capecitabine	Trastuzumab + capecitabine	CTx + HER2	PFS, RR
00073528	ER/HER2+	3	Lapatinib + Letrozole	Placebo + Letrozole	CTx (aromatase-i) + HER2	RR, PFS, safety

CSF: cerebrospinal fluid; CTx: chemotherapy; i: inhibitor; MTD: maximum tolerated dose; NCT-ID: Clinical Trials.gov identifier; OS: overall survival; RR: response rate; PARP: poly (ADP-ribose) polymerase; PDL1: programmed death-ligand 1; PFS: progression-free survival; RTK: receptor tyrosine kinase; SRS: stereotactic radiosurgery; trastuz: trastuzumab; WBRT: whole brain radiotherapy; XRT: radiotherapy; [18F]FLT PET: 3-deoxy-3-18F-fluorothymidine positron emission tomography; HER2: human epidermal growth factor receptor 2; VEGFR: vascular endothelial growth factor receptor.

**Table 2:** A catalogue of brain metastasis genomic studies.

Study	BCBM Only?	Matched Pairs?	Cohort Size	FF or FFPE	GEX	CNA	Mutation Analysis	Exome	WGS	Targeted or Discovery	Key Findings
Bos 2009 [89]	Yes	No	1 *	F	Array	No	No	No	No	D	COX2, HBEGF (EGFR ligand), ST6GALNAC5 (a 2,6-sialyltransferase) over-expressed, mediating BC cell passage through the BBB, with ST6GALNAC5 expression enhancing BC cell adhesion to brain endothelial cells
da Silva 2010 [73]	No	Some	78	FFPE	DASL (512 genes)	No	OncoCarta	No	No	T/D	Over-expression of ≥1 HER, esp HER3 (relative to matched primary tumours); Somatic mutations in *EGFR*, *HRAS*, *KRAS*, *NRAS*, *PIK3CA*; Increased activation of MAPK pathway in BM vs. primary tumours
Ding 2010 [90]	Yes	Yes	1	FF	No	SNP	No	No	Yes	D	Matched peripheral blood, primary tumour, BM and PdX; BM: 2 private mutations, a large deletion, 20 enriched mutations (PdX similar); 2 overlapping large deletions (*CTNNA1*) in all 3 tumour samples; Variation frequencies indicate metastases arise from a minority of cells in the BC
Wikman 2012 [30]	Yes	Some	25	FF	in silico	aCGH/AI	GSS	No	No	T/D	9 loci with significant differences, incl. *EGFR* amp (7p11.2) & 10q22.3-qter loss; AI at *PTEN* more frequent in BM (52%) and brain relapsing BC (59%) compared with BC without relapse (18%; *p* = 0.003) or relapse other than brain (12%; *p* = 0.006); Loss of *PTEN* was especially frequent in HER2-negative BM (64%); *PTEN* mRNA was suppressed in BM compared with primary tumours; *PTEN* mutations were frequently found in BM
McMullin 2014 [91]	Yes	No	19	FF	Array	No	GSS	No	No	T/D	BRCA1 deficient-like GEX signature in HER2+ BCBM in absence of *BRCA1* mutations; Values significantly higher in HER2-/ER- vs. HER2+/ER+ and HER2-/ER+ tumours
Salhia 2014 [32]	Yes	No	35	FF	Array	aCGH ^	No	No	No	D	Frequent large gains 1q, 5p, 8q, 11q, 20q; broad-level deletions (8p, 17p, 21p, Xq); *ATAD2*, *BRAF*, *DERL1*, *DNMTRB* and *NEK2A* frequently amplified & overexpressed; *ATM*, *CRYAB* and *HSPB2* commonly deleted & down-regulated Enrichment in cell cycle and G2/M pathways (incl. *AURKA*, *AURKB* & *FOXM1*; Defects in cell migration and adhesion due to hypermethylation + suppression of *PENK*, *EDN3* and *ITGAM*; Hypomethylation + induction of *KRT8* likely affects adhesion and permeability
Bollig-Fischer 2015 [92]	Yes	No	10	FF & FFPE	No	aCGH	No	No	No	T/D	Stem cell pluripotency pathway enrichment; Recurring amplification of *SOX2*, *PIK3CA*, *NTRK1*, *GNAS*, *CTNNB1*, & *FGFR1*
Brastianos 2015 [93]	No	Yes	86	FF & FFPE	No	No	No	Yes	No	D	86 trios: matched BM, primary tumours, & normal tissue 53% cases had potentially clinically informative alterations in BM; Individual BM deposits genetically homogenous; Distal extracranial and regional node metastases highly divergent from BM; Alterations associated with PI3K/AKT/mTOR, CDK, & HER2/EGFRi sensitivity in BM
Lee 2015 [94]	Yes	Some	42	FFPE	No	No	Ion AmpliSeq Cancer	No	No	T	Frequent somatic mutations (e.g., *TP53* 59.5%, *MLH1* 14.3%, *PIK3CA* 14.3%, *KIT* 7.1%); No significant differences in mutation profiles between BCBM and BC; *TP53* mutation frequency higher in BCBM than in primary BC (59.5% vs. 38.9%)
Saunus 2015 [29]	No	No	36	FF	RNASeq	SNP	No	Yes	No	D	Novel candidate genes: significantly mutated *DSC2*, *ST7*, *PIK3R1* and *SMC5*; DNA repair, HER signalling, axon guidance & protein kinase-A signalling pathways; Potentially actionable genomic alterations in 31/36 BMs (86%); Altered patient management (+trastuz) in a case of HER2 status conversion; *ERBB2* expression correlated with *ERBB3* (*p* < 0.0001); HER3 & HER4 frequently activated in a cohort of 167 BM (7 primary cancer types); HER3 ligands *NRG1/2* barely detectable by RNAseq, with *NRG1* (8p12) genomic loss in 63.6% BCBM, suggesting a microenvironmental source of ligand; Mutational signature analysis facilitated identification of primary type for two CUP
Vareslija 2015 [95]	Yes	Yes	7	U	RNASeq	No	No	No	No	D	ER-specific metastatic pathways; Common pathways altered incl. ECM, adhesion & neuronal differentiation; *ANTRX1*, *THBS2*, *FAP*, *VCAN* & *TIMP2* (invasion/migration/extravasation; EMT/stemness signalling driven by *ANTRX1*; WNT-driven *RUNX* prominent in cells acquiring migration ability
Lee 2016 [96]	Yes	Some	41	FFPE	Nanostring (252 genes)	No	No	No	No	T	22/252 genes differentially expressed between BC and BCBM; *CXCL12*, *MMP2*, *MMP11*, *VCAM1* & *MME* higher in BC, *SOX2* & *OLIG2* higher in BM; PAM50 molecular subtype conversion observed in 8/17 pairs (47.1%)

* pleural effusion sample with subsequent in vivo selection for brain seeking derivatives; ^ study also performed whole genome methylation analysis using the Infinium Human Methylation 27 Bead Array; AI: allelic imbalance; BCBM: breast cancer brain metastasis; BM: Brain metastasis; CNA: copy-number alteration; CUP: cancer of unknown primary; DASL: cDNA-mediated annealing, selection, extension and ligation; ECM, extracellular matrix; FF: fresh-frozen; FFPE: formalin-fixed and paraffin-embedded; GEX: gene expression profiling; GSS: Gene Specific Sanger Sequencing; T: targeted; D: discovery; T/D: elements of both (i.e., study limited by panel, no alternate option at the time); U: unclear; WGS: whole genome sequencing.
